# Fifteen-year trend in treatment outcomes among patients with pulmonary smear-positive tuberculosis and its determinants in Arsi Zone, Central Ethiopia

**DOI:** 10.3402/gha.v7.25382

**Published:** 2014-09-19

**Authors:** Shallo D. Hamusse, Meaza Demissie, Dejene Teshome, Bernt Lindtjørn

**Affiliations:** 1 Oromia Regional Health Bureau, Addis Ababa, Ethiopia; 2 Addis Continental Institute of Public Health, Addis Ababa, Ethiopia; 3 Centre for International Health, University of Bergen, Norway

**Keywords:** tuberculosis, trends, treatment outcomes, Arsi Zone, Ethiopia

## Abstract

**Background:**

Directly Observed Treatment Short course (DOTS) strategy is aimed at diagnosing 70% of infectious tuberculosis (TB) and curing 85% of it. Arsi Zone of Ethiopia piloted DOTS strategy in 1992. Since then, the trend in treatment outcomes in general and at district-level in particular has not been assessed. The aim of this study was to analyse the trend in TB treatment outcomes and audit district-level treatment outcomes in the 25 districts of Arsi Zone.

**Design:**

A retrospective cohort study design was employed to audit pulmonary smear-positive (PTB + ) patients registered between 1997 and 2011. Demographic and related data were collected from the TB unit registers between January and March 2013. The 15-year trend in treatment outcomes among PTB+ patients and district-level treatment outcomes was computed.

**Results:**

From 14,221 evaluated PTB+ cases, 11,888 (83.6%) were successfully treated. The treatment success rate (TSR) varied from 69.3 to 92.5%, defaulter rate from 2.5 to 21.6%, death rate from 1.6 to 11.1%, and failure rate from 0 to 3.6% across the 25 districts of the zone. The trend in TSR increased from 61 to 91% with the increase of population DOTS coverage from 18 to 70%. There was a declining trend in defaulter rate from 29.9 to 2.1% and death rate from 8.8 to 5.4% over 15 years. Patients aged 25–49 years (Adjusted Odd Ratio (AOR), 0.23; 95% CI: 0.21–0.26) and ≥50 years (AOR, 0.43; 95% CI: 0.32–0.59), re-treatment cases (AOR, 0.61; 0.41, 0.67), and TB/HIV co-infection cases (AOR, 0.45; 95% CI: 0.31–0.53) were associated with unsuccessful treatment outcomes.

**Conclusions:**

DOTS expansion and improving population DOTS coverage in Arsi has led to a significant increase in treatment success and decrease in death and defaulter rates. However, there is a major variation in treatment outcomes across the 25 districts of the zone, so district-specific intervention strategy needs to be considered. The low TSR among re-treatment cases might be due to the high rate of MDR-TB among this group, and the issue needs to be further investigated to identify the extent of the problem.

Although effective treatment has been used to treat tuberculosis (TB) for several decades, TB remains a major global health problem and the second leading cause of death worldwide. In 2010, from the estimated 8.8 million incidents of TB cases, 5.7 million TB cases and 1.4 million deaths were reported globally (1, 2). It was also found out that poor adherence and irregular TB treatment leads to an increase in the period of infection with the consequences of multi-drug resistance TB (MDR-TB) (3, 4).

The World Health Organization (WHO) recommended Directly Observed Treatment Short course (DOTS) (5, 6), which aims at detecting 70% of infectious cases and curing 85% of them. This strategy is expected to interrupt transmission of the disease and reduce the number of infected individuals and the period of infectiousness (4).

In 2011, Ethiopia ranked seventh among the 22 high burden countries in terms of estimated number of TB cases (7, 8). Moreover, TB is the third leading cause of hospital admission and the second cause of deaths in Ethiopia (7, 9). In 1992, DOTS strategy started in Ethiopia where Arsi and Bale zones of Oromia region were the first two to be selected for piloting. Since 1997, DOTS has been scaled up to include the entire country (10).

Different studies from southern and northern parts of Ethiopia show that the implementation and expansion of DOTS strategy improved the TB treatment success rate (TSR) and reduced defaulter rates (5, 11–13). Nevertheless, a study from northern Ethiopia indicated that TSR under DOTS strategy was low with high proportion of deaths and defaulters (14).

Although Arsi was the first zone to start DOTS strategy in Ethiopia, no investigation has been made to see if there are differences in TB treatment outcomes across the districts in the zone and also the trend in TB treatment outcomes over the years. Few studies analysed the trends in treatment outcomes as aggregate at either regional or national level. However, to the knowledge of the authors, none of these studies investigated district-specific treatment outcomes to see if there is variation in treatment outcomes across districts in the country (5, 11, 12, 14, 15). Hence, the aim of this study was to analyse trends in TB treatment outcomes over 5 years in the 25 districts of Arsi Zone and to investigate if there were differences in the outcome across the districts.

## Methods

### Study setting

The study was conducted in the Arsi Zone, Central Ethiopia. The zone has one hospital and 73 health centres in 25 districts with a total population of 3.1 million. About 89% of the zonal population resided in rural areas. In 2011, about 70% of the population lived within a 10-km radius or at a walking distance of 2 hours from a health institution and thus had access to DOTS service (16). In 2004, the government of Ethiopia, under the health extension package programme (HEP), launched a community-based essential health service to the community. The HEP is implemented through the deployment of health extension workers (HEWs) at a community level (17). A 1-year undergraduate and two new female cadres are deployed as community health workers at every *kebele* (the smallest government administrative unit) with the responsibility of providing essential health services for a population of 5,000. The objective of HEP is to ensure equitable access to health services, prevent major communicable diseases, and promote health in the community. As part of their role in the prevention of communicable diseases, HEWs are trained on how to identify and refer TB suspects, provide health education and treatment, and trace defaulters (18).

Since 1997, DOTS programme, which started piloting in one health centre and a hospital, has significantly expanded and subsequently gained full integration into 74 health facilities at the end of 2011. All health facilities were utilised as TB diagnostic and treatment units used standard TB unit registers from the National Tuberculosis and Leprosy Control Programme (NTLCP) to register TB cases. Since 2008, TB patients were offered services of provider initiated voluntary counselling and testing for HIV.

The TB drugs used in combination were isoniazid (H), rifampicin (R), pyrazinamide (Z), ethambutol (E), streptomycin (S), and thioacetazone (T). However, since 1997 thioacetazone was removed from the system and not used any more. Using the NTLCP guidelines (9), all new patients were treated with RHZ + E or S for 2 months followed by EH or RH for 6 months. The first 2 months of intensive phase treatment were under direct supervision of the health workers. During this phase, with the exception of those who were critically sick, TB patients received treatment on an ambulatory basis. Re-treatment cases were treated with SERHZ for 2 months, ERHZ for another 1 month, and ERH for 5 months (9).

### Study design and data collection

Retrospective cohort study design was employed to audit TB treatment outcomes for PTB+ patients treated in the 25 districts of Arsi Zone. Pulmonary smear-positive TB cases registered between September 1, 1997, and August 31, 2011, for TB treatment in all public health institutions (73 health centres and one hospital) were included in the study. The principal investigator identified all TB unit registers used in each TB treatment unit during the study period. The total number of TB unit registers in each TB treatment unit over the study period was checked against the total number of annually reported TB cases from each TB treatment unit to the district health office to see if there was any missing TB unit register. Indeed, no missing TB unit registers were detected during the study period. After the identification of all TB unit registers from TB treatment units, the principal investigator collected them between January and March 2013. Ten trained data collectors gathered from TB unit registers socio-demographic and related data like type of TB, TB patient category, contact person for tracing, date of treatment initiation, drug regimen, treatment follow-up, follow-up sputum smear microscopic result, HIV status, treatment outcomes, and date DOTS started in each health institution.

### Definitions of terms

Type of TB and treatment outcome were defined according to the NTLCP guideline adopted from WHO and a previous study report (15, 18).

### Measurements

The area of residence, sex, age, HIV status, TB patient category, treatment regimen, and history of contact person for tracing were taken as independent variables and treatment outcomes as dependent variable. The dependent variable (15-year average treatment outcomes) of each district was computed from the sum of annual TSR of 15 years for each district (nominator) divided by 15 (denominator), which is the number of years of the study period. The trend in PTB+ treatment outcomes of the zone was computed from the sum of the annual PTB+ treatment outcomes of the 25 districts in the zone for each year of the study period. Comparison of 15-year average treatment outcomes among districts was also made after computing the 15-year average of treatment outcomes for PTB+ cases for each district in the zone. Moreover, a comparison of treatment outcomes between the new and the re-treated PTB+ cases was also made.

**Fig. 1:**
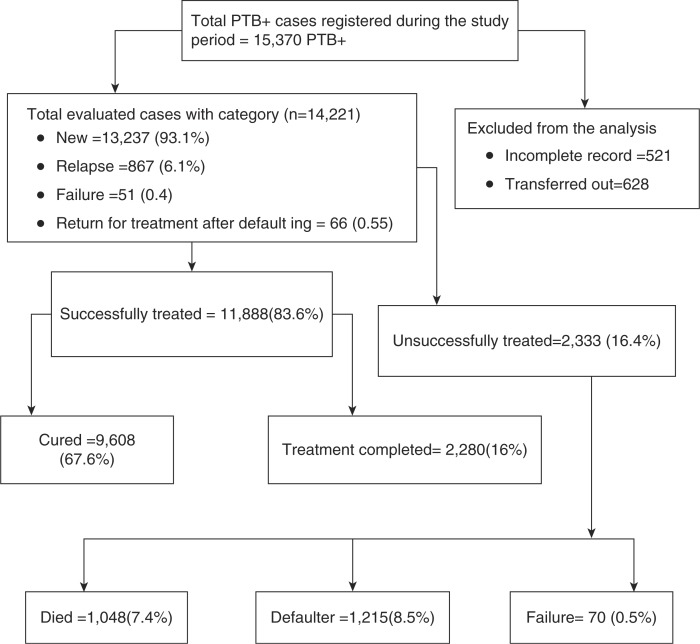
Profile for pulmonary smear-positive TB patients registered during 1997–2011 and treatment outcomes, Arsi Zone, Central Ethiopia.

Population DOTS coverage of the zone was also calculated as the total number of the population residing within a 10-km radius or 2 hours walking distance from a public health institution as nominator and the total number of population of the zone in each year as denominator multiplied by 100. The National Health Service standard where one health centre is expected to serve 25,000 people and one hospital to serve 250,000 people was used to calculate population DOTS coverage (19). A total of 521 PTB+ patients with incomplete records on their treatment information and 628 who were transferred out to other health institutions out of the zone were excluded from the analysis (Fig. 1).

### Statistical analysis

Data were coded and double entered by 10 trained data clerks using Epi-Info version 7. We used IBM SPSS version 20 for data checking, cleaning, and analysis. Descriptive analyses such as frequency, mean, and standard deviation were computed as appropriate. The analyses of linear trend for TSR, cure rate, defaulter rate, and death rate were analysed, and statistical significance was cheeked using *X*^2^ for trend. Bivariate and multivariate logistic regression analysis was used to determine the association between independent and dependent variables. Variables with *p*<0.2 in bivariate analysis were fitted into the final multiple logistic regression models. Variables with *p*<0.05 in the final model were taken as significant determinants. The model adequacy and co-linearity assumptions were checked using F-test and assessed for normality by displaying continuous data on a histogram. All numerical data were found to be normally distributed. Multi-co-linearity of the independent variables was assessed using Pearson correlation, and those with *r* values of 0.6 or less were used in model fitting.

### Ethical approval

An Institutional Review Board of Oromia Regional State Health Bureau, Ethiopia, approved the research for scientific and ethical integrity. Formal permission to use data in the study was obtained from the heads of Zonal and District Health Offices and Health Institutions.

## Results

### Patient characteristics

A total of 15,370 PTB+ patients were registered in 25 districts of Arsi Zone between September 1997 and August 2011. Of these, the treatment outcomes of 14,221 (92.5%) were evaluated. The treatment outcomes of 521 PTB+ patients with incomplete records and 628 transferred out, and hence with unknown treatment outcomes, were excluded from the analysis. From the total evaluated TB cases, 7,734 (54.4%) were males while the remaining were females, and 5,119 (34%) were urban residents. The age of the patients ranged from 1 to 98 years with a mean (standard deviation) of 28.7 (15.3%) years. The majority, 13,237 (93.1%), of the TB patients were new whereas 867 (6.1%) were relapse; 51 (0.4%) were failure; and 66 (0.5%) were return after default. This makes a total of 984 (6.9%) re-treatment cases (Table 1 and Fig. 1).

**Table 1:** General characteristics of pulmonary smear-positive TB cases registered for treatment between 1997 and 2011, Arsi Zone, Central Ethiopia

Characteristics	Number	Percentage
Sex		
Male	7,734	54.4
Female	6,487	45.6
Age group		
0–14	1,939	13.6
15–24	4,389	30.9
25–49	6,207	43.6
≥ 50	1,686	11.9
Mean age (Standard Deviation)	28.7 (15.3)	
Area of residence		
Urban	5,119	34
Rural	9,102	66
Patient category		
New	13,237	93.1
Relapse	867	6.1
Failure	51	0.4
Return for treatment after defaulting	66	0.5
TB/HIV co-infection (*n*=3,627 tested) by type of TB	288	7.9
Treatment outcomes		
Cured	9,608	67.5
Treatment completed	2,280	16
Successfully treated (cured and treatment completed)	11,888	83.6
Died	1,048	7.5
Defaulted	1,215	8.5
Failed	70	0.5

### Trend over time

**Fig. 2:**
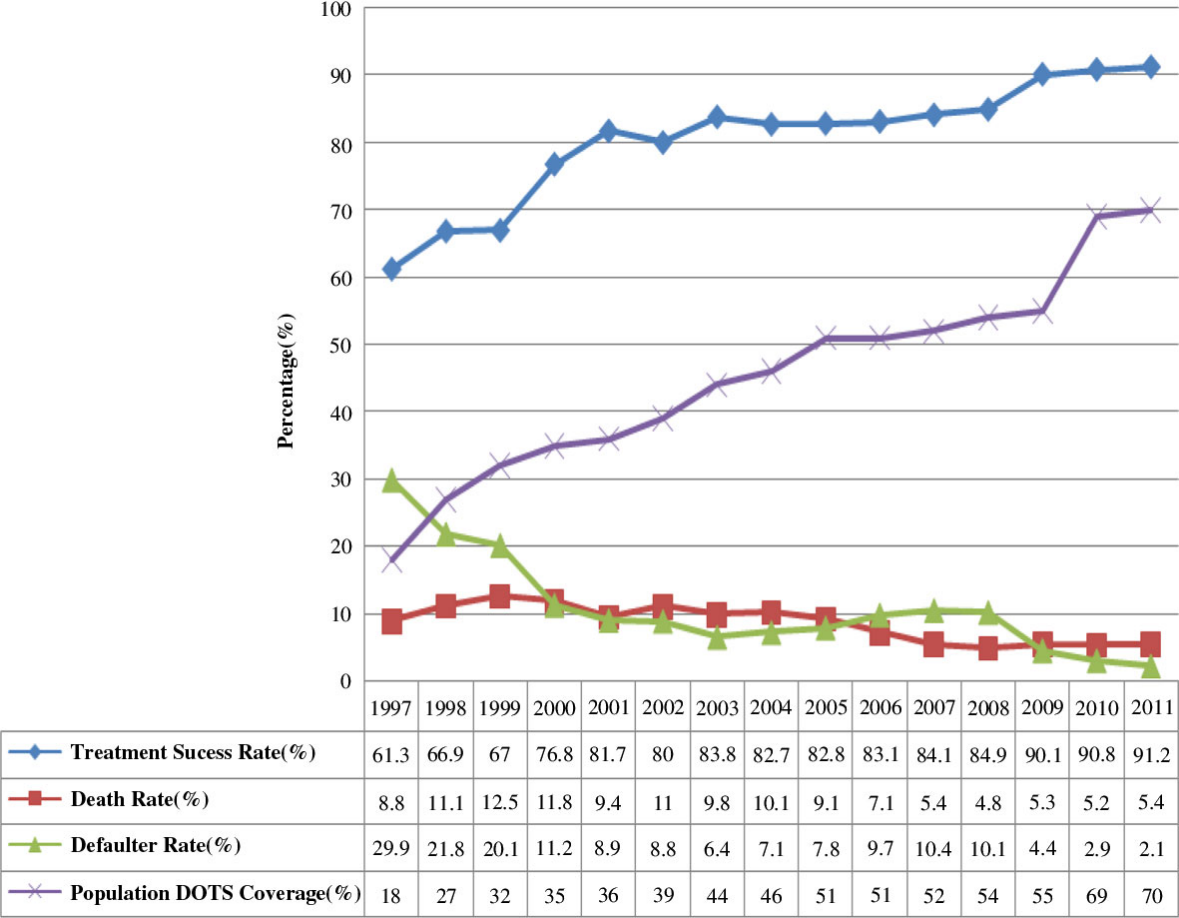
Trends in TB treatment outcomes and population DOTS coverage, Arsi Zone, Central Ethiopia, 1997–2011. *Note*: Trends in treatment success rate of PTB+ increased (*X*^2^trend = 31.08, *p*<0.001) with declined in death rate (*X*^*2*^trend*=*18.56, *p<*0.001) and defaulter rate (*X*^2^trend=33.74, *p*<0.001) over time. Trends in treatment success rate increased; death rate and defaulter rate declined with increase of DOTS population coverage (*X*^2^trend=22.243, *p*<0.001).

The trends in the TB TSR among PTB+ cases increased from 61.3% in 1997 to 91.2% in 2011 with an increase in population DOTS coverage from 18% in 1997 to 70% in 2011. The overall TSR increased by 30% and population DOTS coverage by 52% over 15 years (*X* ^2^ trend=31. 08, *p*<0.001). The TSR and cure rate steadily increased with DOTS site expansion through the years between 1997 and 2001 with the exception of 1999 when a decline was observed. However, the increasing trend over time in TSR stabilised in the range between 82.1 and 84.2% during the years 2003–2008 and then increased to 91.7% in 2011 (Fig. 2).

The trend in death rate among PTB+ cases remained in the high range of 12.5–8.8% during the first 7 years (1997–2005) of DOTS implantation in the study area. The highest death rate (12.5%) was observed in 1999 followed by 11.8% in 2000. However, the trend in death rate gradually declined from 5.7 to 3.9% during the last 5 years of the study period (2008–2011). The trend in defaulter rate also declined steadily from 29.9 to 2.1% over 15 years (1997–2011) (*X*^2^ trend=18.56, *p*<0.001) (Fig. 2).

### Treatment outcomes

The treatment outcomes of 14,221 pulmonary smear-positive TB cases were evaluated. Of these, 11,888 (83.6%) were treated successfully, 9,608 (67.5%) were cured, and 2,333 (16.4%) were treated unsuccessfully. From those treated unsuccessfully, 1,048 (7.4%) died, 1,215 (8.5%) defaulted, and 70 (0.5%) had treatment failure (Table 2).

**Table 2:** Treatment outcomes of new smear-positive and re-treatment pulmonary TB cases of Arsi Zone, Central Ethiopia, 1997–2011

Variable	Treatment outcomes														
	
	Cured		Treatment completed		Successfully treated		Died		Defaulted		Failed		Unsuccessfully treated		
							
	No	%	No	%	No	%	No	%	No	%	No	%	No	%	Total
New (N)	9,085	68.6	2,138	16.2	11,223	84.8	933	7.0	1,026	7.8	55	0.4	2,014	15.2	13,237
Relapse (R)	468	54.0	125	14.4	593	68.4	97	11.2	167	19.3	10	1.2	274	31.6	867
Defaulter (D)	26	39.4	12	18.2	38	57.6	10	15.2	17	25.8	1	1.5	28	42.4	66
Failure (F)	29	56.9	5	9.8	34	66.7	8	15.7	5	9.8	4	7.8	17	33.3	51
Total	9,608	67.6	2,280	16.0	11,888	83.6	1,048	7.4	1,215	8.5	70	0.5	2,333	16.4	14,221
All re-treatment cases (R + D+F)*	523	53.2	142	14.4	665	67.6	115	11.7	189	19.2	15	1.5	319	32.4	984

*Note*: Total cases for each category are the sum of successfully treated and unsuccessfully treated of each category.

* The sum of relapse (R), defaulter (D) and Failure (F).

The TSR was higher (84.8%) among new PTB+ patients compared to re-treatment cases (67.5%). This was mainly due to the high rate of death (11.7%), defaulter (19.2%) and failure (5.6%) among the re-treatment cases. Pulmonary smear-positive new TB cases had higher TSR compared to re-treated PTB+ cases after relapse (84.8% vs. 68.4%); return after default (84.8% vs. 57.6%); and re-treated cases after failure (84.8% vs. 66.7%) (*p<*0.01). On the contrary, death rates were higher among PTB+ cases that returned after default (15.2%); re-treated cases for pervious failure (15.7%); and re-treated cases for previous relapse (11.2%) compared to new PTB+ cases (7%) (*p*<0.01) (Table 2).

The defaulter rate among PTB+ cases that returned after defaulting treatment was high (26.6%), and such patients were more likely to default again compared to new PTB+ cases (7.8%) (*p*<0.01). On the other hand, PTB+ patients re-treated for previous failure had higher treatment failure (7.8%) and were more likely to have treatment failure again compared to new PTB+ patients (0.4%) (*p*<0.01) (Table 2).

### Factors associated with unsuccessful TB treatment outcomes

Logistic regression analyses revealed that patients who are urban residents, older ones, re-treated cases, those co-infected with HIV, and those with no contact person were less likely to be treated successfully. In the final model, TB patients in the age group 25–49 (AOR, 0.26; 95% CI: 0.53–0.95) and above 50 years of age (AOR, 0.42; 95% CI: 0.33–0.60) were less likely to be treated successfully compared to the younger age groups. Moreover, re-treatment cases (AOR, 0.61; 0.41, 0.67) in comparison with new ones, and TB/HIV co-infected cases (AOR, 0.45; 95% CI: 0.31–0.53) in comparison with non-TB/HIV co-infected ones were less likely to be successfully treated (Table 3).

**Table 3:** Factors associated with TB treatment success rate among pulmonary smear-positive TB patients registered during 1997–2011 in Arsi Zone, Central Ethiopia

		Treatment success categories			
				
Variables	Category	Not successfully treated	Successfully treated	COR (95% CI)	AOR (95% CI)
Patients’ residence	Urban	794 (15.5)	4,325 (84.5)	1.00	1.00
	Rural	1,138 (12.5)	7,964 (87.5)	1.49 (1.40,1.58)**	1.13 (0.96,1.37)
Age	0–14 years	192 (9.9)	1,747 (90.1)	1.00	1.00
	15–24 years	421 (9.6)	3,968 (90.4)	1.06 (0.95,1.21)	1.14 (0.84,1.53)
	25–49 years	1006 (16.2)	5,201 (83.8)	0.59 (0.52,0.68)**	0.74 (0.53,0.95)**
	≥50 years	325 (19.3)	1,361 (81.7)	0.47 (0.44,0.56)**	0.42 (0.35,0.60)**
Sex	Male	1,060 (13.7)	6,674 (86.3)	1.00	1.00
	Female	895 (13.8)	5,592 (86.9)	1.05 (0.98,1.11)	1.10 (0.94,1.30)
Patient category	New	2,014 (15.2)	11,223 (84.8)	1.00	1.00
	Re-treatment	319 (32.4)	665 (67.6)	0.47 (0.43,0.56)**	0.61 (0.41,0.67)**
HIV status	HIV −ve	227 (6.8)	3,112 (93.2)	1.00	1.00
	HIV +ve	49 (17.1)	239 (82.9)	0.35 (0.34,0.47)**	0.45 (0.31,0.53)**
Having, contact person	Yes	1,770 (13.3)	11,537 (86.7)	1.00	1.00
	No	158 (17.0)	756 (83.0)	0.76 (0.68,0.85)**	0.87 (0.61,1.35)

** *Note*: Significant at *p*<0.001.

### Treatment outcomes among PTB+ cases by district

The overall treatment outcomes among PTB+ cases registered for treatment during the study period varied across the 25 districts of the zone (*X*^2^=317. 35, *p*<0.001). The 15-year average TSR varied from 69.3 to 92.5%. Defaulter rate ranged between 2.5 and 21.6%, whereas death rate spanned from 1.6 to 11.1%. It was also observed that districts with low TSR (69.3%) had high rates of default (16.9%), death (10.2%), and failure (3.6%) while those with high treatment success (more than 85%) had low rates of default and death. Moreover, high death rate was observed in districts that had high TB/HIV co-infection (Table 4).

**Table 4:** Treatment outcomes of all pulmonary smear-positive (new plus re-treated cases) TB patients by districts in Arsi Zone, Central Ethiopia, 1997–2011

Districts	Treatment outcomes for smear-positive pulmonary TB (*N*=14221)					TB/HIV co-infection		
	
	Total PTB +	Cured rate	Treatment success	Defaulter rate	Death rate	Failure rate	Number of tested	HIV positive

	*N*	*N* (%)	*N* (%)	*N* (%)	*N* (%)	*N* (%)	*N*	*N* (%)
Tiyo	131	58 (44.3)	113 (86.3)	12 (9.2)	4 (3.1)	2 (1.5)	42	4 (9.5)
Amigna	162	86 (52.4)	117 (72.2)	35 (21.6)	9 (5.6)	1 (0.6)	73	5 (6.8)
Aseko	373	259 (66.9)	310 (83.1)	41 (11.0)	21 (5.6)	1 (0.3)	138	2 (1.4)
Assala town	1,591	1,244 (76.7)	1,334 (83.8)	117 (7.4)	131 (8.2)	9 (0.6)	372	86 (23.1)
Ble Gezegar	256	128 (49.6)	214 (83.6)	24 (9.4)	18 (7.0)	0 (0.0)	58	4 (6.9)
Cholle	408	302 (72.8)	357 (87.5)	26 (6.4)	24 (5.9)	1 (0.2)	162	9 (5.6)
Digalutijo	970	715 (72.4)	829 (85.5)	55 (5.7)	79 (8.1)	7 (0.7)	247	16 (6.5)
Diksis	399	193 (47.9)	309 (77.9)	64 (16)	21 (5.3)	5 (1.3)	72	1 (1.4)
Dodota	1,425	911 (63.9)	1,149 (80.6)	108 (7.6)	158 (11.1)	5 (0.4)	180	36 (20)
Gololcha	444	303 (67.9)	401 (90.3)	35 (7.9)	7 (1.6)	1 (0.2)	222	5 (2.3)
Guna	466	238 (50.6)	344 (73.8)	80 (17.2)	40 (8.6)	2 (0.4)	98	2 (2)
Hetosa	840	570 (66.9)	688 (81.9)	84 (10.2)	66 (7.9)	2 (0.2)	218	20 (9.2)
H/Wabe	170	74 (43.3)	152 (89.4)	9 (5.3)	8 (4.7)	1 (0.6)	58	1 (1.7)
Jaju	961	730 (74.0)	821 (85.4)	59 (6.1)	77 (8.0)	4 (0.4)	253	11 (4.3)
L/Bilbilo	918	695 (74.1)	796 (86.7)	59 (6.4)	61 (6.6)	2 (0.2)	173	10 (5.8)
L/Hitosa	604	455 (73.6)	542 (89.7)	23 (3.8)	37 (6.1)	2 (0.3)	165	17 (10.3)
Merti	644	428 (65.5)	517 (80.3)	64 (9.9)	62 (9.6)	1 (0.2)	146	15 (10, 3)
Munesa	323	213 (65.1)	268 (83.0)	36 (11.1)	15 (4.6)	4 (1.2)	180	8 (4, 4)
Robe	996	657 (65.4)	821 (82.4)	92 (9.2)	78 (7.8)	5 (0.5)	169	14 (8.3)
Shirka	580	360 (61.6)	468 (80.7)	71 (12.1)	40 (6.9)	2 (0.3)	117	4 (3.4)
Sire	525	363 (68.5)	437 (83.2)	54 (10.3)	30 (5.7)	4 (0.8)	108	4 (3.7)
Sude	387	201 (51.3)	350 (90.4)	19 (4.9)	18 (4.7)	0 (0.0)	120	1 (0, 8)
Tena	166	98 (57.0)	115 (69.3)	28 (16.9)	17 (10.2)	6 (3.6)	68	5 (7.4)
Z/Dugda	320	233 (72.6)	296 (92.5)	8 (2.5)	15 (4.7)	1 (0.3)	101	3 (3)
Seru	167	91 (53.5)	140 (83.8)	13 (7.6)	12 (7.2)	2 (1.2)	87	5 (5.7)
Total	14,221	9,608 (67.5)	11,888 (83.6)	1,215 (8.5)	1,048 (7.4)	70 (0.5)	3,627	288 (7.9)

*Note*: Total cases include those under treatment success, defaulter, death and failure rates. L/Bilbilo: Lemu Bilbilo; L/Hitosa: Lode Hitosa; Z/Dugda: Zuway Dugda.

## Discussion

Expanding DOTS and improving its population coverage has increased TSR in PTB+ cases and has led to improved overall TB treatment outcomes in the study area. There are poor treatment outcomes among re-treatment cases compared to the new ones. Further, there are significant differences in TB treatment outcomes among the 25 districts of the zone. These findings might help the TB control programme managers and policy makers to look for an alternative strategy for addressing the inequality in TB treatment outcomes across different geographical settings in the country.

In this study, TB TSR increased significantly from 61.3 to 91.2% and parallel with the expansion of DOTS population coverage from 18 to 70% in 15 years. The upward trend in TSR was inversely proportional to the declining trend in defaulters which went from 29.9 to 2.1% and deaths from 12.5 to 5.4% over time. Such a result goes with findings by other studies that showed a similar trend of increase in treatment success and decline in death and default rate (5, 11, 20). Moreover, the increase in TSR during the last 3 years of the study period was significant and higher than the 85% target recommended by WHO.

The TSR increased by 31% through the improvement of population DOTS coverage by 52% in 15 years. This is higher than the 13.4% rise in TSR and the 44% increase in population DOTS coverage over 7 years in southern Ethiopia (11). It is also higher than the 15% increase in TSR over 14 years in Vietnam (20) and the 18% global average increase gained in a 10-year period (21). The increase in trend observed during the last 3 years of the present study is significant and could be attributed to the stepwise deployment of HEWs at community level. Since 2008, it has been possible to cover all *kebeles* through two female HEWs (19). The involvement of HEWs in a TB control programme might have improved access and played a significant role in the reduction of defaulter cases. This implies the importance of further decentralisation of DOTS service to the community level where there are resource constraints and limited access to health services to achieve MDG in TB control.

The overall 15-year average TSR among PTB+ patients was 83.6%. This is high compared to findings by previous studies which showed 29.5 and 28.3% in northwest Ethiopia (14, 22), 55.7% in western Ethiopia (23), and 77% in Tanzania (24). However, it is lower than the 88% success rate in China (25) and 89% in southern and northern Ethiopia (12, 26). The difference could be due to variation in the length of the study period and the sample size across different study areas. It could also be due to variation in the study setting. Majority of the patients in northern Ethiopia were from urban centres, and about 25% of them were HIV co-infected (14, 22) compared to the 7.9% in the current study (15). Obviously, TB/HIV co-infection is associated with poor treatment outcome (27). In addition, TB cases transferred out in large numbers in northern Ethiopia were included in the analysis, whereas the actual treatment outcome of this group is not known. Thus, their inclusion in the computation would influence the report on TSR (13, 14, 22).

The TSR among re-treatment cases is much lower than new PTB+ cases. This confirms previous studies where re-treatment cases were significantly associated with unsuccessful treatment outcome (11, 12, 28, 29). Moreover, the current study revealed that there is high death rate and failure rate among re-treatment cases compared to the new ones. WHO recommended a microbial culture and drug susceptibility test (DST) for all re-treatment TB cases and new PTB+ cases who failed to convert sputum examination results at the end of a second month of follow-up (30). However, due to limited access to culture and DST services in Ethiopia, the services were not provided for MDR suspected cases; hence, the extent of MDR among re-treatment cases was not known. Moreover, the high death and failure rate in this group in the current study might also be due to high prevalence of MDR-TB in the group (28, 29). Thus, the findings of the study might warrant further investigation to determine the extent of MDR-TB in Ethiopia. This could help in giving a quick response to the current global challenge of MDR-TB.

Reports from elsewhere revealed that the prevalence of MDR-TB was more than 10-fold among previously treated patients than untreated cases (28). Study results from northern Ethiopia also indicated that previous anti-TB drug exposure had 6.4 times risk of developing MDR-TB compared to new TB cases (31). The prevalence of MDR-TB is estimated to be between 1.6 and 1.8% among new and between 12 and 18% among re-treatment cases in Ethiopia (8, 32). Establishing microbial culture and DST services at least at referral hospital to detect MDR-TB cases as early as possible and initiating appropriate treatment is an urgent issue to be addressed in Ethiopia.

The overall 8.6% 15-year average defaulter rate in the current study was lower than reports from elsewhere in and outside the country (10, 33–35). However, there are also other studies with lower rates than the rate in this study (22, 23) and the global WHO target of less than 5%. Patients returning for re-treatment after defaulting were much more likely to default again (26.6%) compared to new patients (7.0%). This finding is in line with previous reports where such patients were more likely to default again compared to new cases (11, 12). Poor adherence to anti-TB treatment due to defaulting and irregular treatment may lead to more severe illness, treatment failure, relapse, longer infection, drug resistance, and even death. Thus, defaulting and irregular intake of anti-TB drugs are a challenge and concern for the individual patient as well as for the community and they need to be addressed properly.

The average 15-year failure rate (0.5%) in the present study corroborates previous reports of 0.2, 0.5 and 0.3% failure rate in northwest and western Ethiopia (14, 22, 23). However, it is lower than the 1.2% rate among new PTB+ and the 6.4% among re-treatment cases in southern Ethiopia (11), the 3.2% among new PTB+ in north Ethiopia (12) and the 2.8% in China (25). The large number of transfer outs and defaulters in the present study might have exerted an influence on the failure rate as it might be higher if the transfer outs were evaluated and defaulters completed their treatment.

The present study indicated that TB patients in the age range of 25–49 and above 50 years, and those co-infected with HIV were independently associated with unsuccessful TB treatment outcome. This is supported by other studies (11, 12, 33, 35, 36) where similar results were observed. On the contrary, sex, area of residence, and patient category showed no significant association with unsuccessful TB treatment outcome, and this is inconsistent with findings from other studies as well (11, 13, 22, 35).

Findings from this study also illustrate a significant variation in treatment outcomes among patients across the 25 districts of the zone. The highest TSR (92.5%) with lowest defaulter (2.5%) and failure (0.3%) rates in Zuway Dugda district might indicate the role of effective TB treatment in the reduction of defaulter cases and drug-resistant strain. However, the lowest TSR (69.3%) with highest failure (3.6%), defaulter (17%), and death (10.2%) rates in Tena district might reflect the consequences of poor TB treatment (3, 4).

The high death rate observed among districts with high TB/HIV co-infection in the current study substantiates previous reports of high mortality rate among TB/HIV co-infected cases (3, 37). Overall, the variation in treatment success, defaulter, death, and failure rates across districts of the zone could be due to the real differences in DOTS performance and disparity in quality of TB control programme (11). Thus, the study warrants TB programme managers and policy makers to identify locality specific challenges to be addressed in order to universally achieve the global WHO recommended rate of 85% treatment success.

Although the study has established the usefulness of facility-based data analysis of a 15-year period, it has some limitations. This retrospective facility-based study lacks inclusion of patients’ important variables such as educational level, knowledge about TB, duration of the treatment, distance from the treatment centre, family size, family support, medication side effect, and income that have been reported to have an association with TB treatment outcomes (12, 13, 33). Because of the inherent limitations of a retrospective study, incomplete data were excluded from the analysis and this might affect the results of this study. Moreover, counting all patients who were treated with a full course of anti-TB drugs but with missing records on their treatment outcomes as defaulters could introduce bias as these patients might have completed the treatment, died, or failed.

## Conclusions

DOTS expansion and improving population DOTS coverage in Arsi has led to a significant increase in treatment success and a decrease in death and defaulter rates. However, there is a major variation in treatment outcomes across the 25 districts of the zone, so a district-specific intervention strategy needs to be considered. The low TSR among re-treatment cases might be due to a high rate of MDR-TB among this group, and the issue needs to be further investigated to identify the extent of the problem.
